# Supplementary material for: HSF1 Can Prevent Inflammation following Heat Shock by Inhibiting the Excessive Activation of the ATF3 and JUN&FOS Genes
Source: Cells. 2022 Aug 12;11(16):2510. doi: 10.3390/cells11162510 (PMC9406379; doi:10.3390/cells11162510)
Supplement: Supplementary file 1 [file cells-11-02510-s001.zip › cells-1819492-supplementary/Figure S1-S9.pdf]

Supplementary Figures

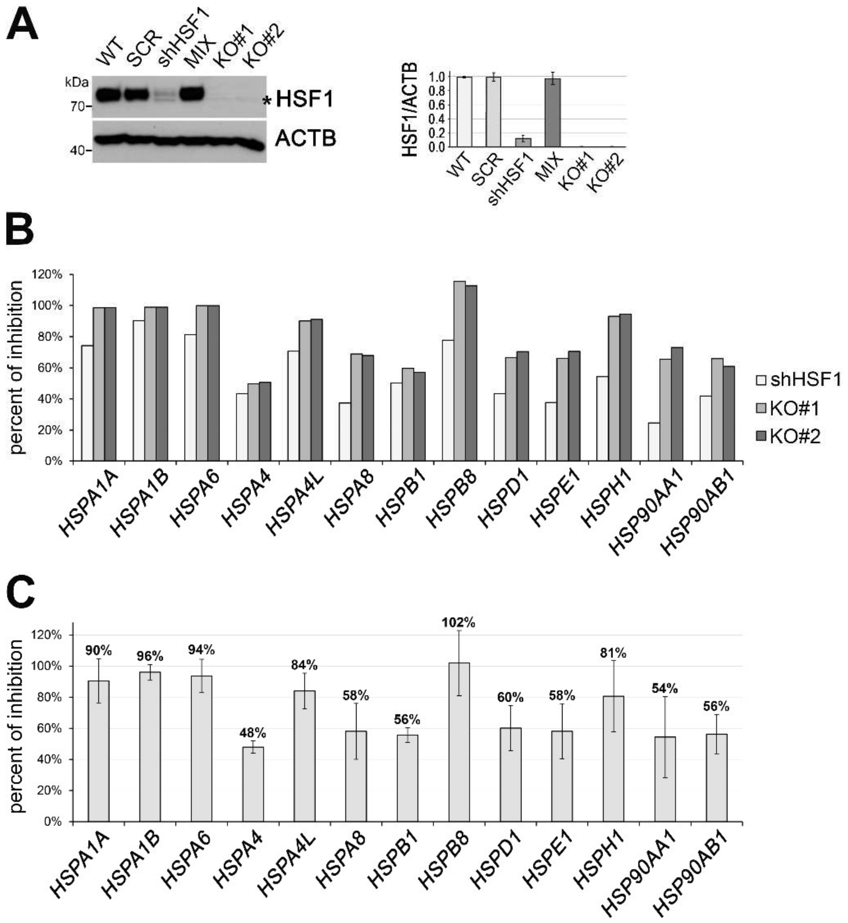

Figure S1. Effect of HSF1 deficiency on heat-inducibility of selected *HSP* genes. (**A**) Western blot analysis of HSF1 level in MCF7 cell variants used for RNA-seq analyses: unmodified cells (WT), variants stably transduced with non-specific shRNA (SCR) or with HSF1-specific shRNA (shHSF1), and a combination of control clones (MIX) or two HSF1-negative clones (KO#1, KO#2) arisen from single cells following CRISPR/Cas9 gene targeting. The asterisk shows non-specific bands. Actin (ACTB) was used as a protein loading control. The graph on the right shows the results of densitometric analyses of HSF1 immunodetection (n=3). WT, SCR, and MIX cells are referred to as HSF1-proficient (HSF1^{prof}) while shHSF1, KO#1, and KO#2 as HSF1-deficient (HSF1^{def}). (**B** and **C**) The degree of inhibition of activation by heat shock of selected *HSP* genes in HSF1 deficient cells (data extracted from RNA-seq): shown separately (B) or as the mean (C) for all HSF1-deficient lines. Note that only the HSF1 knockout resulted in almost complete inhibition of the induction by heat shock of some *HSP* genes, while others were only partially inhibited.

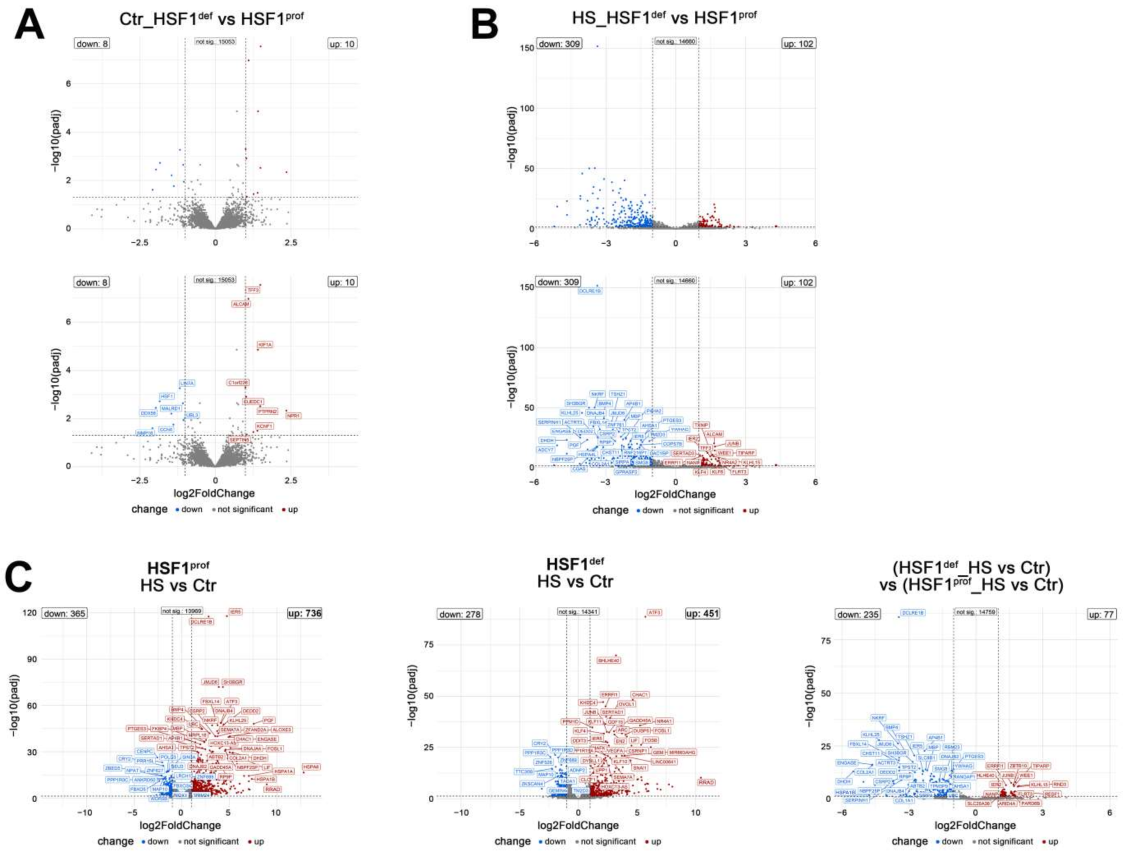

Figure S2. Volcano plots of RNA-seq results showing the differentially expressed genes in (**A**) untreated control (Ctr) and (**B**) heat-shocked (HS) cells (cells with reduced versus normal levels of HSF1, HSF1− vs HSF1+, were compared). Upper plots – without gene labels, lower plots – with gene labels. (**C**) Volcano plots from Figure 1A-C but with gene labels showing the differentially expressed genes in response to heat shock (HS) in cells with normal and reduced levels of HSF1 (HSF1+ and HSF1−, respectively), and a comparison of the response in both cell variants.

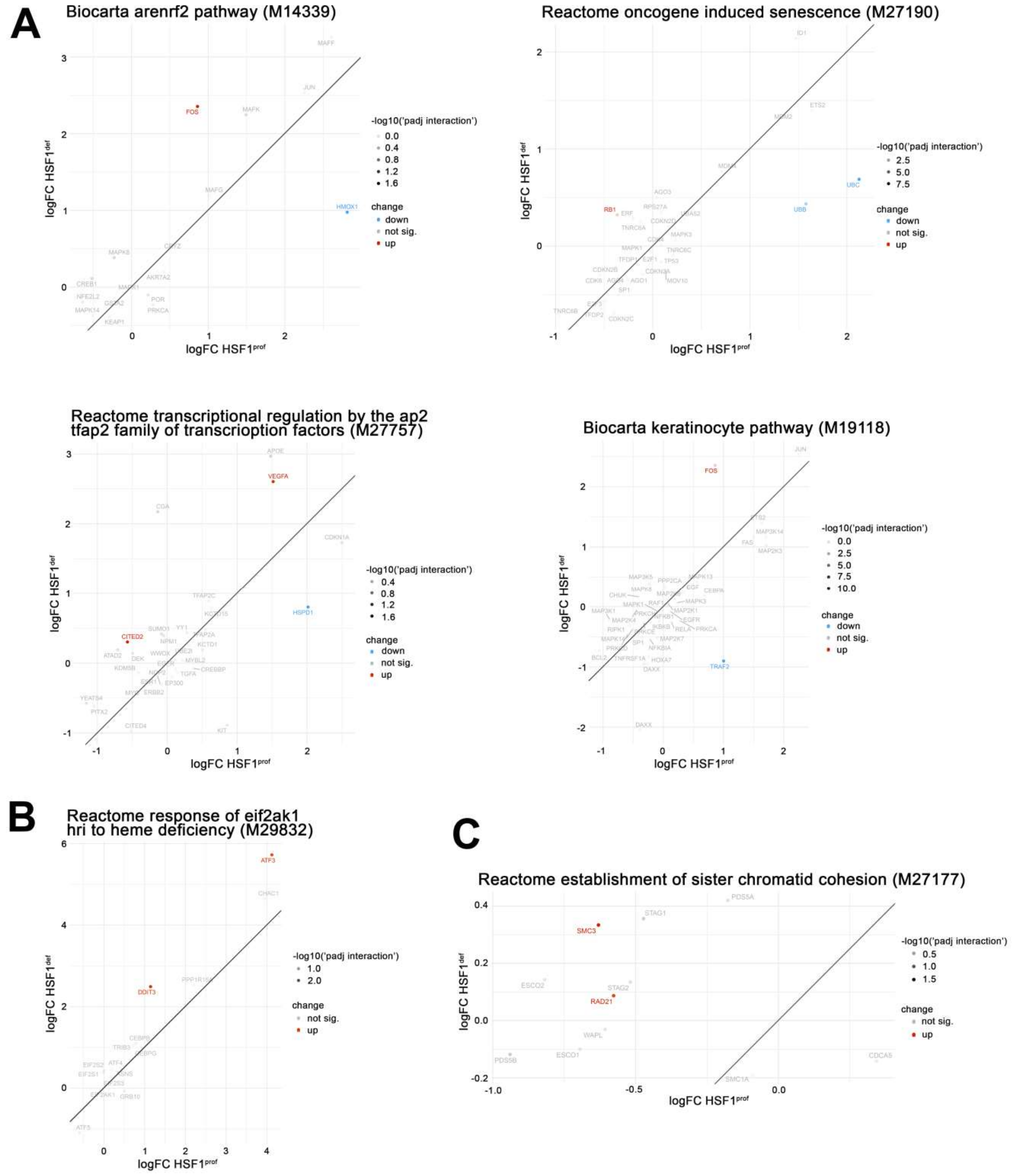

Figure S3. Scatterplots of log2-fold-changes upon heat shock treatment in HSF1-proficient (on X-axis) and HSF1-deficient (Y-axis) cells. Genes associated with selected genesets (shown in Figure 1D) enriched in: (**A**) HSF1^{def} cells only, (**B**) both HSF1^{prof} and HSF1^{def}, and (**C**) differentiating HSF1^{def} and HSF1^{prof} cells.

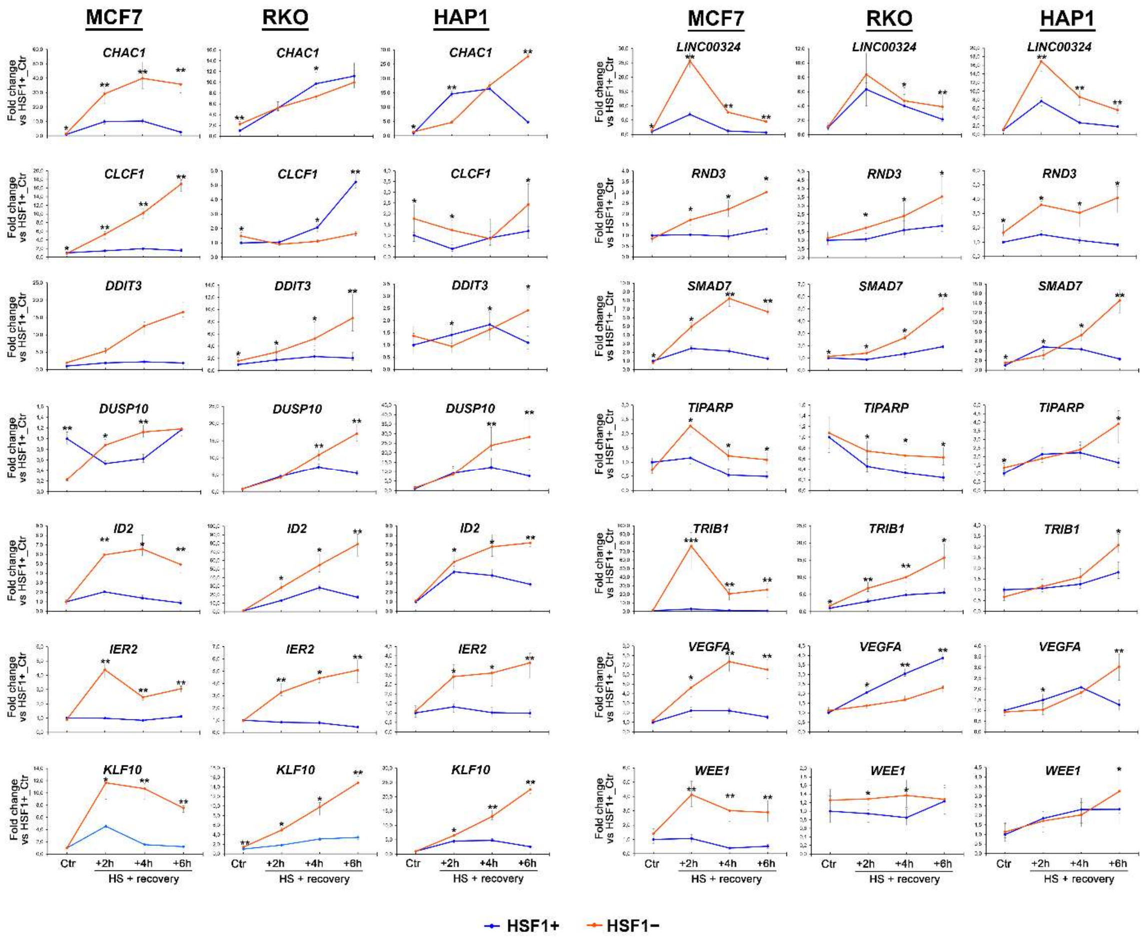

Figure S4. Transcriptional response to heat shock in HSF1+ and HSF1− MCF7, HAP1, and RKO cells. Expression of indicated genes analyzed by RT-qPCR in cells exposed to elevated temperature (HS: 43 °C/1h + recovery 37 °C/2h, 4h, or 6h) in relation (fold change) to untreated control (Ctr) in HSF1+ cells. In the case of MCF7, a different cell model was used than for RNA-seq. The difference between HSF1+ and HSF1− in each time point: **p < 0.001, *p < 0.05.

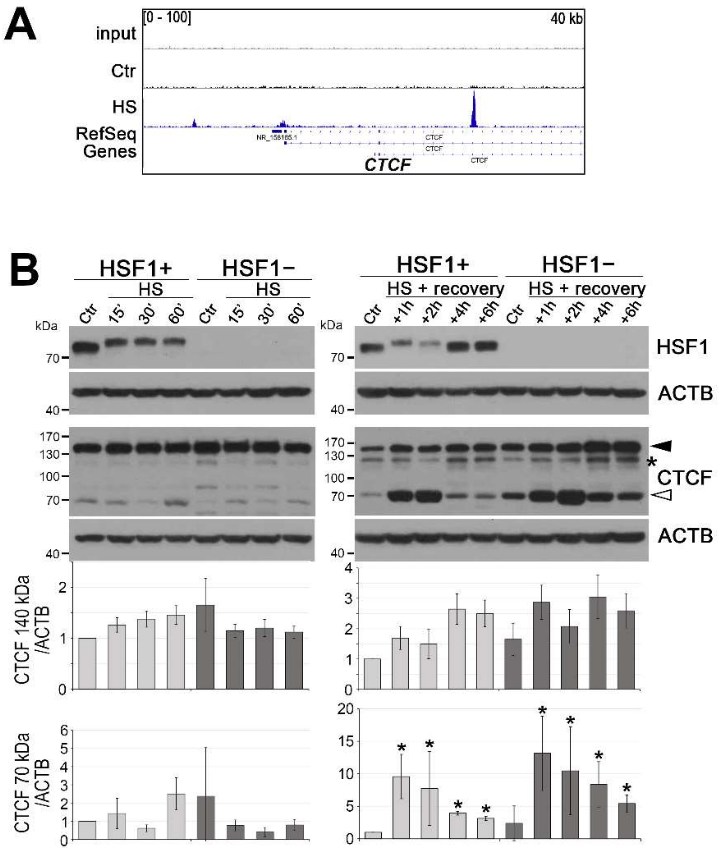

Figure S5. Possible involvement of HSF1 in the regulation of CTCF expression following heat shock in MCF7 cells. (**A**) HSF1 peaks in the *CTCF* gene identified in ChIP-seq analyses and visualized by the IGV browser in untreated cells (Ctr) and after heat shock (HS: 15 min at 43 °C). The scale is displayed in the left corner, length of the region shown – in the right corner. (**B**) Western blot analyses of HSF1 and CTCF in HSF1+ and HSF1− cells, untreated and after 15 – 60 min heat shock at 43 °C (left) or after one hour heat shock and 1-6 hours of recovery (right). ACTB was used as the loading control. The black arrowhead shows the protein bands reported as CTCF (above 130 kDa), white arrowhead – C-terminally truncated CTCF (70 kDa). The asterisk shows non-specific bands detected by the anti-CTCF Ab (Diagenode; 1:4,000). The graph below shows the results of densitometric analyses of CTCF immunodetection (n=2-3, depending on experimental point). Statistically significant differences between HS and corresponding Ctr: $*p < 0.05$.

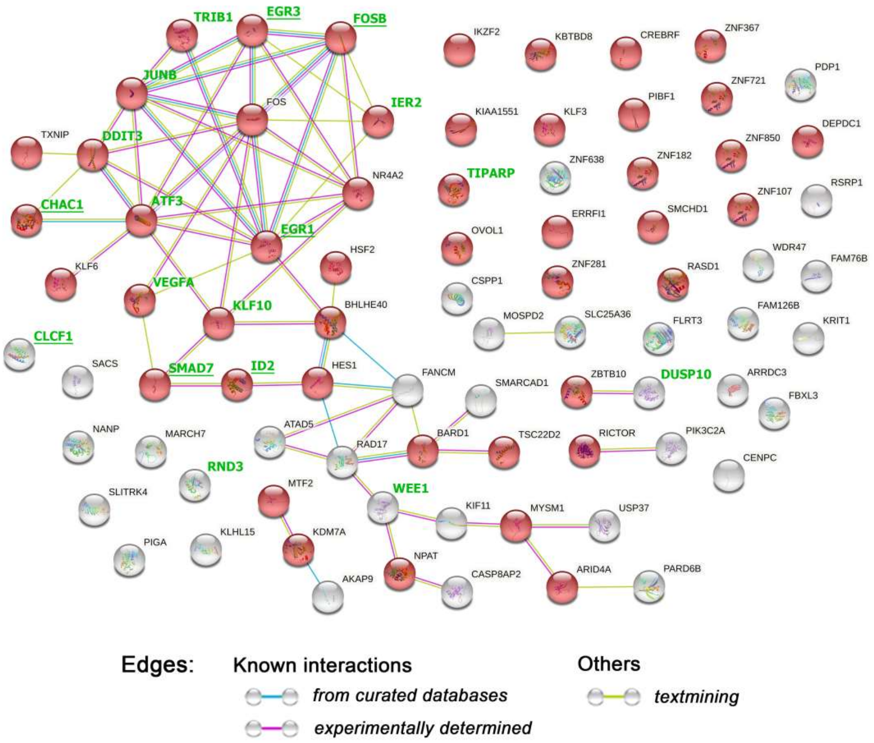

Figure S6. The network of interactions between proteins encoded by genes that were either more strongly induced by heat shock in HSF1-deficient cells or not as strongly repressed as in HSF1-proficient cells. Known protein-protein interactions were depicted using STRING version 11.5 (three types of links with the confidence threshold of 0.4 are shown). Functional enrichment in the regulation of gene expression (GO:0010468; FDR = 1.07e-07) is presented as nodes in shades of red. Symbols of genes selected for RT-qPCR validation are in green (the upregulated genes additionally included in the analysis, apart from the group of 77 genes from Figure 1C, are underlined).

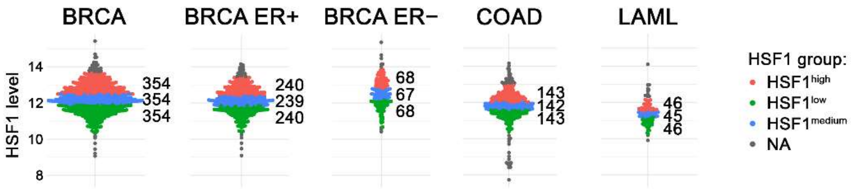

Figure S7. Selection of cases with different levels of HSF1 expression and group sizes in TCGA Breast Invasive Carcinoma (BRCA), Colon Adenocarcinoma (COAD), and Acute Myeloid Leukemia (LAML).

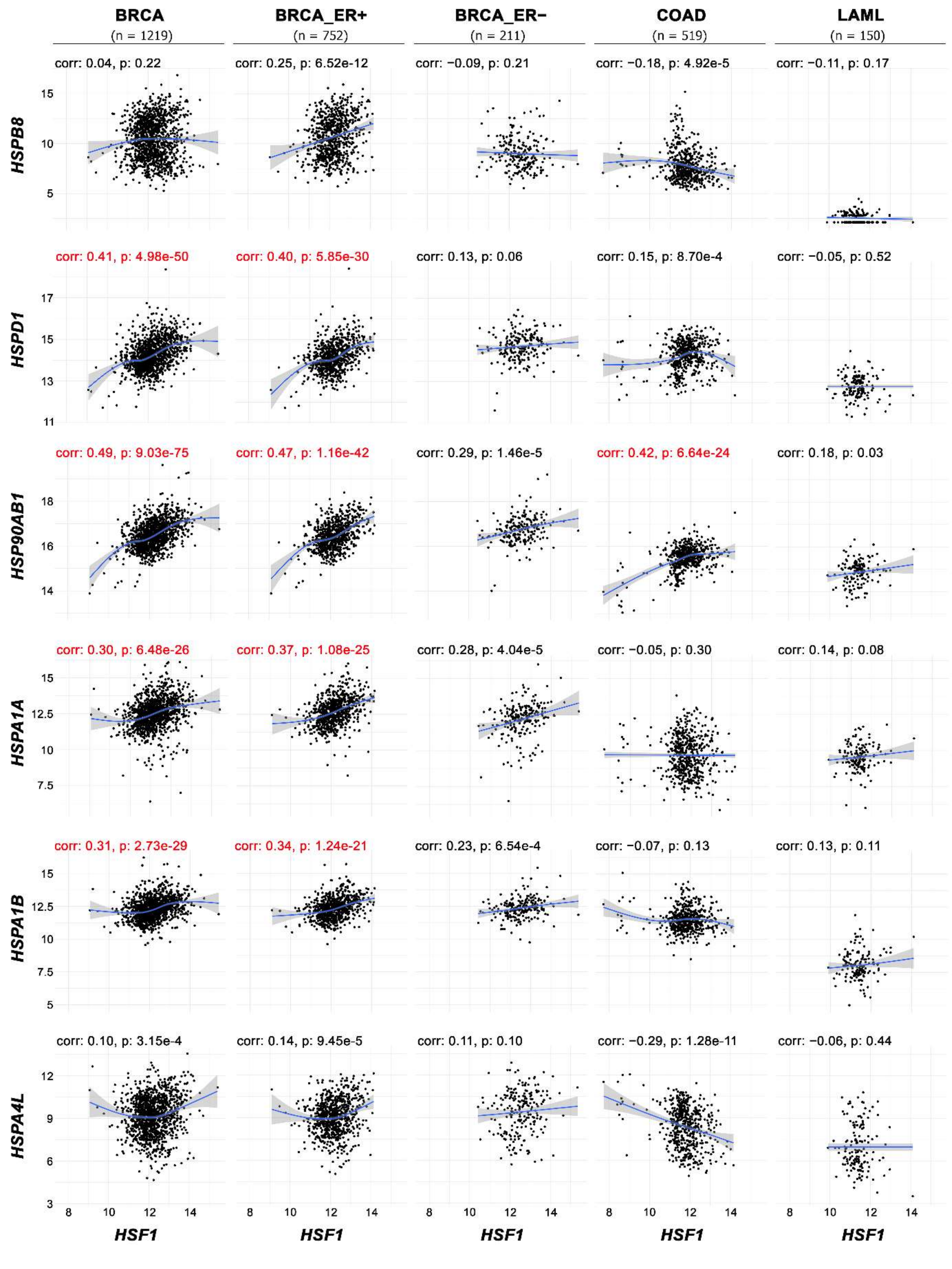

Figure S8. Correlation of *HSF1* and selected *HSPs* transcript levels (normalized counts) in TCGA Breast Invasive Carcinoma (BRCA; ER+ and ER−, estrogen receptor-positive and negative, respectively), Colon Adenocarcinoma (COAD), and Acute Myeloid Leukemia (LAML). Each dot represents one cancer case. Correlation coefficients > 0.3 are marked in red.

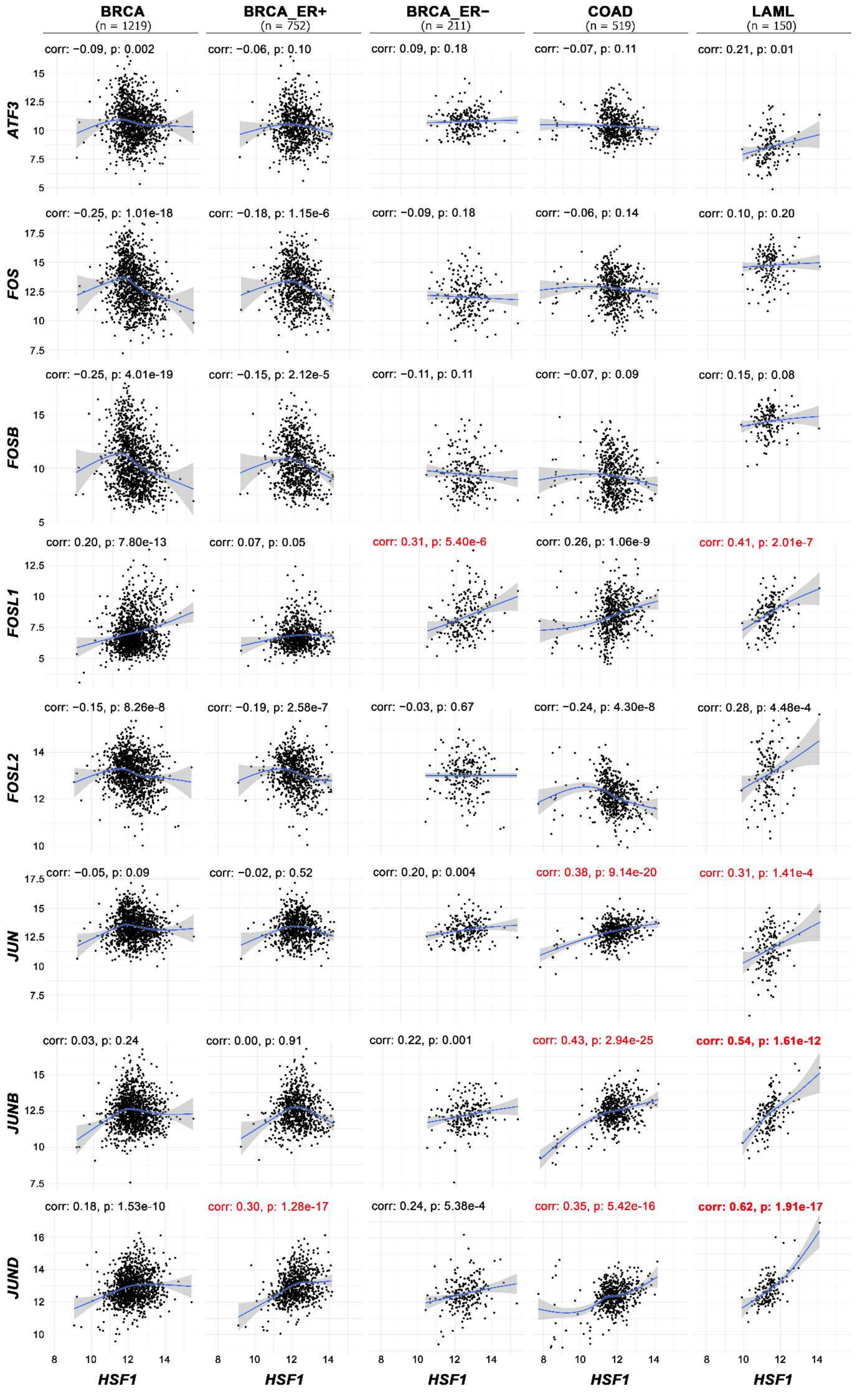
BRCA
(n = 1219)
BRCA_ER+
(n = 752)
BRCA_ER−
(n = 211)
COAD
(n = 519)
LAML
(n = 150)
ATF3
corr: −0.09, p: 0.002
corr: −0.06, p: 0.10
corr: 0.09, p: 0.18
corr: −0.07, p: 0.11
corr: 0.21, p: 0.01
FOS
corr: −0.25, p: 1.01e-18
corr: −0.18, p: 1.15e-6
corr: −0.09, p: 0.18
corr: −0.06, p: 0.14
corr: 0.10, p: 0.20
FOSB
corr: −0.25, p: 4.01e-19
corr: −0.15, p: 2.12e-5
corr: −0.11, p: 0.11
corr: −0.07, p: 0.09
corr: 0.15, p: 0.08
FOSL1
corr: 0.20, p: 7.80e-13
corr: 0.07, p: 0.05
corr: 0.31, p: 5.40e-6
corr: 0.26, p: 1.06e-9
corr: 0.41, p: 2.01e-7
FOSL2
corr: −0.15, p: 8.26e-8
corr: −0.19, p: 2.58e-7
corr: −0.03, p: 0.67
corr: −0.24, p: 4.30e-8
corr: 0.28, p: 4.48e-4
JUN
corr: −0.05, p: 0.09
corr: −0.02, p: 0.52
corr: 0.20, p: 0.004
corr: 0.38, p: 9.14e-20
corr: 0.31, p: 1.41e-4
JUNB
corr: 0.03, p: 0.24
corr: 0.00, p: 0.91
corr: 0.22, p: 0.001
corr: 0.43, p: 2.94e-25
corr: 0.54, p: 1.61e-12
JUND
corr: 0.18, p: 1.53e-10
corr: 0.30, p: 1.28e-17
corr: 0.24, p: 5.38e-4
corr: 0.35, p: 5.42e-16
corr: 0.62, p: 1.91e-17
HSF1
HSF1
HSF1
HSF1
HSF1

Figure S9. Correlation of transcript levels (normalized counts) of *HSF1* and *ATF3*, *JUN* and *FOS* family members in TCGA Breast Invasive Carcinoma (BRCA; ER+ and ER−, estrogen receptor-positive and negative, respectively), Colon Adenocarcinoma (COAD), and Acute Myeloid Leukemia (LAML). Each dot represents one cancer case. Correlation coefficients > 0.3 are marked in red.
