# Supplementary material for: HSF1 Can Prevent Inflammation following Heat Shock by Inhibiting the Excessive Activation of the ATF3 and JUN&FOS Genes
Source: Cells. 2022 Aug 12;11(16):2510. doi: 10.3390/cells11162510 (PMC9406379; doi:10.3390/cells11162510)
Supplement: Supplementary file 1 [file cells-11-02510-s001.zip › cells-1819492-supplementary/Table S1-S2.pdf]

Table S1. RT-qPCR primers for gene expression analyses.

Gene symbol	RefSeq	forward primer sequence	reverse primer sequence
ATF3	NM_001206486.2	cctctgtggcatcaccagggtt	tgcctggatgcaggggtgta
CHAC1	NM_001142776.4	tctccggccacaaccttgaa	tgtccacatgagcactcccc
CLCF1	NM_001166212.2	gcttggctgggacctatctga	cattgaggcttcgccacacc
DDIT3	NM_001195057.1	tgggtagtgtggcccaagtg	cgactcgccgagctctgatt
DUSP10	NM_007207.6	ggctgaacatcggctacgtc	ctggtgagcttcctcaatgaac
EGR1	NM_001964.3	gcacctgaccgcagagtctttt	actgaccaagctgaagagggg
EGR3	NM_001199880.1	aacgagaagcccaacccggaa	aggtcacggtcttgttgccg
FOSB	NM_001114171.2	gaggtacagcggcatcctgt	taaggtgcaagtccggggtg
HSPA1A	NM_005345.5	agctggagcaggtgtgtaaccc	aaaaacagcaatcttggaaaggccc
ID2	NM_002166.5	cctgcagcacgtcatcgacta	agaagggaattcagaagcctgc
IER2	NM_004907.3	caatgagtgtttggccgcga	cgctcacgcctctctacaca
IL6	NM_000600.5	taccccccaggagaagattcc	ttttctgccagtgcctcttt
JUNB	NM_002229.3	acacagctacgggatacggc	gtccgagccctgaccagaaa
KLF10	NM_001032282.4	acaatcccagcattttgtttgac	gtgcggcaatgtgaggtttg
LINC00324	NR_026951.1	atgcgcagttttgccacagg	tcctcagtagaaggataggagggg
RND3	NM_001254738.1	tgggacacttcgggttctcc	tcagacttgcagccgaccaa
SMAD7	NM_001190821.2	aagtccgccacactggacaa	atctgcacggtaaagcccgt
TIPARP	NM_001184717.1	aagccaactctcggggtctg	ggaaccccaccaagtgtctgta
TNF	NM_000594.4	ttctcgaaccccgagtgaca	gccttggcccttgaagagga
TRIB1	NM_001282985.2	ctcctatgtgcgaagccgga	agctgggttctctcctccgt
VEGFA	NM_001025366.3	atcctgtgtgcccctgatgc	tcctatgtgctggccttggt
WEE1	NM_001143976.2	gttggccgaggcttgaggta	tgttacatgcccaagatcacctat
ACTB	NM_001101.5	agagcctcgcctttgccgat	ttgcacatgccggagccgtt
GAPDH	NM_002046.7	ttccatggcaccgtcaaggc	tgcaaatgagccccagccttct
HNRNPK	NM_002140.4	atgctgtcctcattccactgac	cgcgacggtcatcaaacatca
HPRT1	NM_000194.3	gccctggcgtcgtgattagt	tgatggcctcccatctcctt

Table S2. ChIP-qPCR primers for HSF1 and HSF2 binding analyses.

Gene symbol	RefSeq	forward primer sequence	reverse primer sequence
ATF3 (1)	NC_000001.11	cagctcctgctcggatttcagc	gaagtttcttcctggacggcgac
ATF3 (2)	NC_000001.11	agcttcacgtgttctccctcc	cgggattaggtggaggagtgt
HSPD1/HSPE1	NC_000002.12	ccggcttagtctagttcccg	cgatttgacccttgagccgt
HSPH1	NC_000013.11	tgcccattgggtagaatctttc	gaggtcccacttcctcagcctta
JUN	NC_000001.11	atagcccatgatgtcaccccaag	cagcggagcattacctcatccc
JUND	NC_000019.10	tgacgtccgtaagtttcctgg	tcctctctccccaacaactcg
FOSB	NC_000019.10	tcgggtcccagacctgaagcta	aggaaaggaaaagagactgctggaa
FOSL1	NC_000011.10	cctaggggacatgcctgcaa	gcttagcttaccctcgccca
negative control locus	NC_000012.12	atggttgccactggggatct	tgccaaagcctaggggaaga
